# Melting Behavior of Heterogeneous Polymer Bulk Solids Related to Flood Fed Single Screw Extruders

**DOI:** 10.3390/polym12122893

**Published:** 2020-12-02

**Authors:** Christian Kneidinger, Erik Schroecker, Gernot Zitzenbacher, Jürgen Miethlinger

**Affiliations:** 1 School of Engineering, University of Applied Sciences Upper Austria, Stelzhamerstraße 23, 4600 Wels, Austria; Erik.Schroecker@fh-wels.at (E.S.); G.Zitzenbacher@fh-wels.at (G.Z.); 2 Institute of Polymer Extrusion and Compounding, Johannes Kepler University of Linz, Altenbergerstraße 69, 4040 Linz, Austria; juergen.miethlinger@gmail.com

**Keywords:** single screw extruder, melting, melting experiment, model experiments, heterogeneous materials, mixtures, bends

## Abstract

Melting models for flood fed single screw extruders, like the Tadmor model, describe the melting of pure thermoplastic polymers. However, the melting behavior of heterogenous polymer systems is of great interest for recycling issues, for example. In this work, the melting of polymer mixtures and that of pure bulk polymers by the drag induced melt removal principle is examined both theoretically and experimentally. The applied model experiments represent the melting of the solid bed at the barrel in single screw extruders. As polymer pellet mixtures, polypropylene-homopolymer mixed with polypropylene-block-copolymer, high density polyethylene, polyamide 6, and polymethylmethacrylate were studied using different mixing ratios. The melting rate and the shear stress in the melt film were evaluated dependent on the mixing ratio. The results show that when processing unfavorable material combinations, both shear stress and melting rate can be far below that of pure materials, which was also confirmed by screw extrusion and screw pull-out experiments. Furthermore, approaches predicting the achievable melting rate and the achievable shear stress of polymer mixtures based on the corresponding values of the pure materials are presented.

## 1. Introduction

Due to their outstanding price-performance ratio and their reliability, single screw extruders (SSE) are used in polymer processing whenever possible. The modelling of these extruders is commonly subdivided into functional zones, which are the hopper, the flow-in zone, the solids conveying zone, the delay zone, the melting zone, and the metering zone. In some cases, additional mixing and degassing zones are used [1,2,3,4,5,6,7,8,9]. The throughput, the quality, and the homogeneity of the processed material are strongly influenced by the melting performance of SSE.

**Figure 1:**
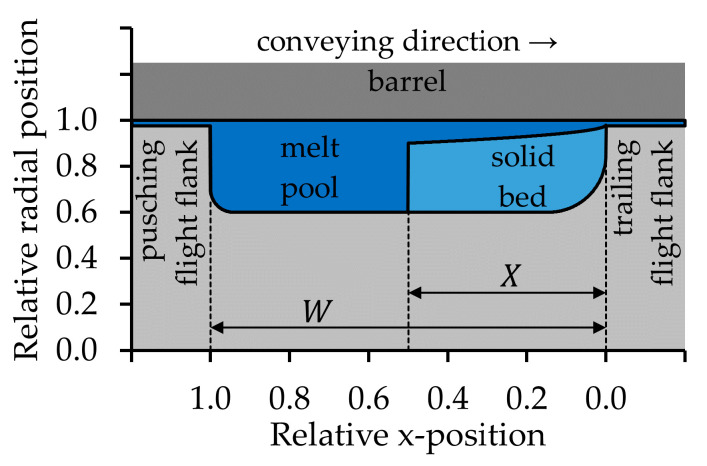
Simplified schematic drawing of the contiguous solids melting mechanism (CSM) in the flat screw channel, adopted and adapted from Gogos and Tadmor [6]. W is the channel width and X is the width of the solid bed. The melt film between the solid bed and both the screw root and the passive screw flight is not shown.

The typical melting process in flood fed SSE was first observed by Maddock [10]. The reported mechanism is valid in flood fed SSE when materials are processed which do not tend to slip on the wall. Most SSE are flood fed SSE and most polymer types are wall-adhering polymers. At the beginning of the melting process, a melt film develops between the barrel and the compacted solid bed, and later a thin melt film surrounds the solid bed. Then, the melt film at the barrel is scraped off by the active screw flight flank. The melt is cumulated in a melt pool in front of the active screw flight flank. The solid bed is pressed towards the passive screw flight flank, as Figure 1 shows. The solid bed gradually decreases while it is transported through the melting zone. Maddock [10] stated, that the biggest amount of material is melted between the solid bed and the heated barrel. Agassant et al. [5], Rauwendaal [2,11], and White and Potente [7] called this mechanism “contiguous solids melting” (CSM), while Chung [12] referred to it as “dissipative melting”.

Klenk [13], Gale [14], and Chung [15] observed another melting mechanism. Contrary to the melting mechanism described by Maddock [10], the melt pool was formed between the solid bed and the passive screw flight. Klenk and Gale processed polyvinyl chloride (PVC) and concluded that wall slip was responsible for this melting behavior. Later Chung showed that this mechanism can also occur when the flight clearance is too large. Lindt [16] observed melt pools on both sides of the solid bed while processing polypropylene (PP) in an extruder with a screw diameter of 90 mm. Dekker [17] and Chung [15] reported the same mechanism in the same year.

In co-rotating twin screw extruders, the solid pellets and the melted material are mixed, which results in a faster melting behavior as the solid fragments are surrounded by melted polymer. This melting mechanism is named “dispersed solids melting” (DSM) by Agassant et al. [5], Rauwendaal [2,11], and White and Potente [7]. Chung [12] referred to it as “conduction melting”. Zhu and Chen [18], Huang and Peng [19], and Rauwendaal [2,11,20,21] reported that the DSM mechanism can appear in single screw extruders with a specific screw design. Rauwendaal [11] demonstrated that about half of the material must be melted before DSM can occur. Furthermore, Rauwendaal [11,21] and Huang and Peng [19] compared DSM and CSM and found DSM to be much more efficient.

Wilczyński et al. [22,23] reported that polymer blends are widely used in industry, but that their melting behavior is substantially different to that of pure polymers. This finding is also confirmed by Cunha et al. [24,25,26]. Furthermore, a lot of post-consumer waste, which in general consists of a mixture of different polymeric materials, has to be recycled. Wilczyński et al. [27,28] studied the melting behavior and the morphology development of polymer mixtures in a single screw extruder with a screw diameter of 45 mm and compared it to the melting behavior of the pure materials. The mixtures were composed of low-density polyethylene (LDPE) and polystyrene (PS) with an LDPE concentration of 85 % and 95 %, respectively. They reported that the melting mechanism of mixtures is much more complicated than that of pure PS. Moreover, they found that the plastication of PS was delayed to a much greater distance along the screw compared to the pure material. Tyagi, Domingues, Lindt, Wilczyński et al. [22,23,27,28,29,30,31,32,33,34] published papers dealing with the morphology development of polymer blends in single screw extruders. They pointed out, that the compatibility of the polymers (surface tension, thermodynamically behavior, and the viscosity ratio) and the deformation (shear, elongation) are important parameters for the development of the morphology. Cunha et al. [24,25,26] studied the melting of PP/ PA6 mixtures in a single screw extruder and showed that both mechanisms (CSM, DSM) can exist at the same time. They observed a mechanism that combines Maddocks CSM mechanism, while solid particles distributed in the melt pool were melted by the DSM mechanism. They called this mechanism the “hybrid melting mechanism” (HMM). Wilczyński et al. [22,23,27,32,35,36,37] observed the same mechanism when processing polymer mixtures in flood feed SSE. They described it as a “CSM mechanism with an obvious presence of minor component inclusions”. Gale [38] treated compounding with single-screw extruders and also investigated mixtures of plastics with different melting points. He found that it can happen that the material with a higher melting point is transported through the extruder without being sheared.

Wilczyński et al. [22,23,27,32,35,36,37] reported, that in starve fed SSE the polymer mixture is melted much faster via the DSM mechanism. Furthermore, Wilczyński et al. [23] noted that “a melting model considering the presence of two materials is not currently (2017) available” and proposes applying “simply-structured mixing rules” for the calculation. They focused on starve fed SSE. From this point of view, one should propose to use starve fed single screw extrusion for polymer mixtures. Strand et al. [39] claims that in industrial processes flood feed is usually used in SSE as starve feed reduces the throughput and the pressure build-up.

The “drag induced melt removal model” (Tadmor [40]) is a major component of the modelling of the CSM mechanism in flood fed SSE. In this model, a solid polymeric sample is pressed against a hot moving surface with infinite extension and the melting of the polymer is determined. The model allows the calculation of the melting rate as a function of the velocity and the surface temperature (Gogos and Tadmor [6]). Model experiments based on Tadmor’s drag induced melt removal model showed that the melting behavior of polymer bulk solids is not only dependent on the temperature and the velocity, but also on the shape of the material and the pressure (Kneidinger et al. [41]). Spheroidal pellets melt faster compared to cylindrical ones and their melting rate is more pronounced with rising pressure. During melting more polymer melt penetrates into the free space between spheroidal pellets compared to cylindrical ones (Kneidinger et al. [42]). In addition, cylindrical shaped bulk solids exhibit a higher external coefficient of friction compared to spheroidal ones, which boosts the solid conveying behavior and increases the extruder output (Längauer et al. [8,9]). The coefficient of friction of mixtures of different polymers of investigated by Shim [43].

In this paper, the melting of polymer mixtures in a contiguous solid melting process related to flood fed single screw extrusion is studied, which has not been systematically analyzed in such detail until now. For this purpose, a model experiment based on Tadmor´s drag induced melt removal model is employed to analyze the melting rate and shear stress of different polymer mixtures dependent on the mixing ratio. The device was inspired by works of the groups of Chung, Spalding, Vermeulen, and Sundstrom [15,44,45,46,47,48,49,50,51,52,53]. In addition, a color evaluation method was developed, which can analyze the temporal sequence of the melting of distinct polymers. The obtained melting parameters of the pure materials are compared to an existing calculation method. Mixing rules are proposed as an approach to show if it is possible to predict the melting rate of the polymer mixtures from the melting rate of the pure materials. Furthermore, the results are verified by extrusion experiments and by screw pull-out tests.

## 2. Methods and Experiments

**Figure 2:**
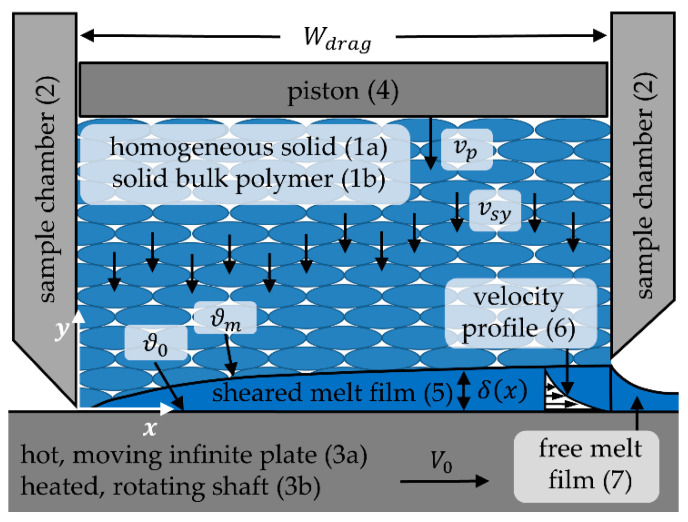
Schematic drawing of the principle of the melting experiment used ($W_{drag}\;$ = width of the solid bed, $v_{p}$ = piston velocity, $v_{sy}$ = moving velocity of the solid, $\vartheta_{0}$ = temperature of the moving plate, $\vartheta_{m}$ = melting temperature, $V_{0}$ = moving velocity of the plate, δ = melt film thickness, x,y = coordinate directions).

In order to investigate the melting behavior of mixtures of polymer bulk solids via the CSM mechanism a model experiment based on Tadmor´s drag induced melt removal model is employed [6]. This model describes the melting of the solid bed on the hot barrel via the CSM mechanism in a SSE (see Figure 2). An infinite isotropic, homogeneous solid slab (1a) of the width $W_{drag}$ is pressed against a hot, moving plate with infinite lateral extension (3a). In the experiment, the isotropic, homogeneous solid is replaced by a solid bulk polymer mixture (1b) that is filled into the sample chamber (2). The infinite moving plate is replaced by a heated rotating shaft (3b), which is equipped with a scraper (not shown here) to remove the melt film. Furthermore, a piston (4) is used to apply pressure upon the bulk. The temperature $\vartheta_{0}$ of the surface is higher than the melting temperature $\vartheta_{m}$ of the polymer. The surface is moving in the x-direction with the velocity $V_{o}$. The solid slab is moving in the negative y-direction at the velocity $v_{sy}$. Due to the temperature dependency of the material density ρ, this velocity is a function of the *x* and *y* -position, or rather a function of the temperature distribution in the entire solid slab. In the experiment, the correlating velocity of the piston in the *y* -direction $v_{p}$ is measured by a very sensitive position measurement system. A melt film of the thickness δ as a function of the *x*-position is formed. This melt film (5) is sheared inside the sample chamber, resulting in a velocity distribution within the melt film (6). The free melt film outside of the sample chamber (7) is not sheared, so the melt film thickness within the sample chamber is larger than after it.

### 2.1. Experimental Setup for the Melting Experiments and for the Bulk Density Measurements

**Figure 3:**
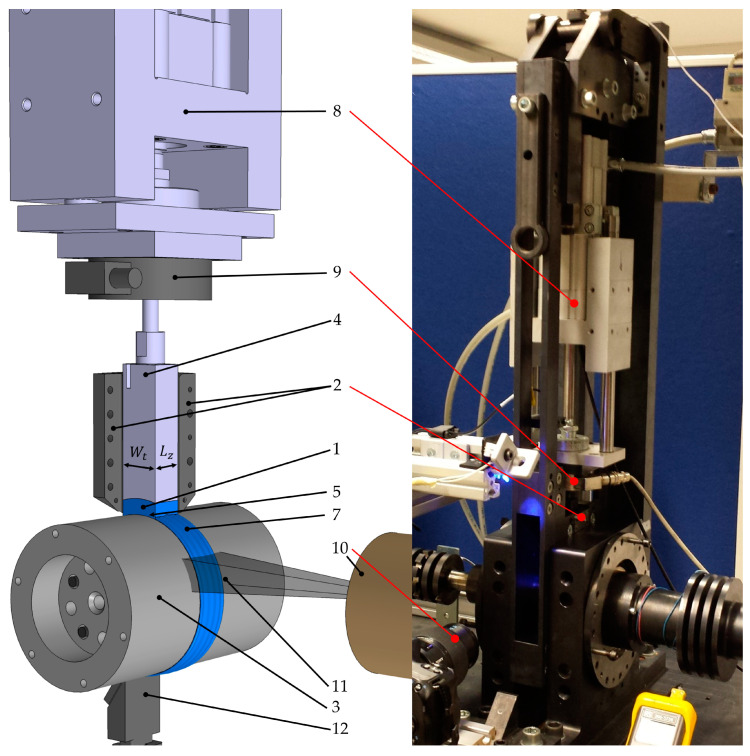
Three-dimensional visualization and photography of the test apparatus (the consecutive numbering is consistent to Figure 2). Polymeric sample (1), sample chamber (2), shaft (3), piston (4), sheared melt film inside the sample chamber (5), melt film outside of the sample chamber (7), piston (8), load cell measuring the normal force (9), macro lens (10), field of view (11), and scraper (12).

A previously developed method ([41,42]) (depicted in Figure 3, the consecutive numbering is consistent to Figure 2 was used to carry out the melting experiments. In addition, the method was enhanced by introducing a color evaluation method to enable the determination of the temporal sequence of melting related to the distinct polymers.

The temperature of the sample chamber (2), the shaft (3), and the piston (4) as well as the applied normal force (4) and the rotational velocity of the shaft (3) can be varied in a wide range. In this manner, conditions close to those in single screw extrusion can be guaranteed. The maximum achievable temperature is 350 °C (662 °F). The pneumatic cylinder (8), which is equipped with a position encoder and a linear guide, generates normal forces ($F_{N}$) of up to 2 kN (450 lbf). The normal force that is applied to the pellets via the piston (4) is measured by a load cell (9) and enables normal stresses of up to 20 MPa (2900 psi). Three different sample chambers and matching pistons with differing cross-sectional areas are available. The surface-velocity $V_{0}$ of the exchangeable shaft can be chosen from 0.04 to 1.5 m/s (1.5 to 59 inch/s). The shaft (3) with a diameter of 100 mm (3.93 inch) is heated using four 100 W heating cartridges. The surface temperature is measured using a thermocouple and managed by a highly developed power control system, guaranteeing constant temperature conditions. The force generated due to the shearing of the melt film is measured via another load cell. The data is recorded at a rate of 250 Hz. The molten polymer forms a melt film, which is sheared (5) inside of the sample chamber. A single-lens translucent camera is used to capture videos in a resolution of 1920 × 1080 pixel at a framerate of 25 frames per second in progressive scan mode. A macro lens (10) with a focal distance of 100 mm enables very detailed videos and photos, as the field of view (11) is very narrow. The recorded video files were analyzed after the experiments to evaluate, if the polymer mixtures are melted simultaneously or subsequently. A scraper (12) is used to scrape the molten polymer from the shaft. In this work, a sample chamber (2) with an axial length ($L_{z}$) of 21 mm (0.83 inch) and tangential width ($W_{t}$) of 27 mm (1.06 inch) and a polished shaft made of the tool steel 1.2311 (40CrMnNiMo8-6-4) were used. The density was measured at room temperature. In the melting experiments, the shaft (3) was heated but not the sample chamber (2) or the piston (3).

### 2.2. Melting Experiments

Before starting the experiments, the shaft is heated to the surface temperature $\vartheta_{0}$, which was 200 °C in the experiments with the polymer mixture of polypropylene homopolymer (PP-H), grade A and high-density polyethylene (HDPE) and 240 °C (464 °F) in all other experiments. The polymer mixture samples are prepared before starting the melting experiment. As an example, 2.5 g HDPE pellets are manually mixed with 2.5 g PP-H pellets. An amount of 5 g of solid bulk polymer mixture (1) is filled into the sample chamber (2). After this, the piston (4) is inserted into the sample chamber (2) and the automated test sequence is started. This is done quickly to avoid a preheating of the bulk solids. The bulk material is pressed upon the heated shaft at 2.0 MPa (290 psi). The shaft rotates at a circumferential velocity of 0.12 m/s (4.7 inch/s). These conditions were chosen after preliminary experiments. Furthermore, the velocity is comparable to the circumferential speed of the lab scale single screw extruder at 120 min^−1^ which was applied to verify the results. In this experiment, the molten polymer is transported out of the sample chamber and forms a melt film. The scraper is pressed onto the shaft to scrape off the melt film. Resulting in preferable constant conditions during the whole experiment. The measured position of the piston (4) and the experimentally determined shear stress is analyzed to characterize the melting behavior.

The measured melting rate per area ($w_{A,meas}$) is calculated from the velocity of the piston ($v_{p}$) and the bulk density ($\rho_{bulk}$) of the material

(1) $$w_{A,meas}=v_{p}\rho_{bulk}.$$

The velocity of the piston ($v_{p}$) is evaluated from the piston position $y_{p}$

(2) $$v_{p}=-\frac{dy_{p}}{dt},$$

where t is the measurement time. The shear stress is measured with a second load cell, which is not shown in the figures. Each test was performed four times consecutively. The calculated standard deviations of the measured values are represented by error bars in the results diagrams.

### 2.3. Bulk Density Measurements

The bulk density values were determined according to an experimental method which was described by Längauer et al. [8]. Five grams of the polymer bulk mixture was filled into the sample chamber. The piston applied an increasing normal stress on the material and the piston position ($y_{p}$) was analyzed to calculate the bulk density ($\rho_{bulk}$)

(3) $$\rho_{bulk}=y_{p}A_{proj}.$$

The projected cross-sectional area ($A_{proj}$) of the sample chamber is calculated from its axial length ($L_{z}$) of 21 mm (0.827 inch) and from its tangential width ($W_{t}$) of 27 mm (1.06 inch)

(4) $$A_{proj}=L_{z}W_{t}\;.$$

Each test was performed four times consecutively and the plotted error bars show the calculated standard deviations of the measured values. The presented bulk densities of the material mixtures were measured at room temperature at a mean normal stress of 2.0 MPa (290 psi).

### 2.4. Extrusion Experiments and Screw-Pullout Experiments

Screw-pullout experiments were conducted utilizing a lab scale smooth bore single screw extruder Type E20M from Collin Lab & Pilot Solutions, Maitenbeth, Germany, with a screw diameter of 20 mm (0.79 inch) and an L/D-ratio of 25. The dimensions of the used three section screw are given in Table 1 and the set temperature profile in Table 2. A slit die with an adjustable die gap height (between 0.1 and 2 mm) and a die gap width of 50 mm was used. The die gap height was set to 0.5 mm in the screw pull-out experiments. The screw speed of the extruder was set to 120 min^−1^, which results in a circumferential speed of 0.126 m/s which is slightly faster than the velocity of the model experiments.

### 2.5. Modelling of the Melting Behavior of Pure Materials

Gogos and Tadmor [6] note that several authors have analyzed this melting problem both theoretically and experimentally. The model of Pearson [54] is summarized here, because it is needed for the calculation of the melting behavior of the pure polymers and to compare those results to the experimentally obtained values. This model was originally developed for semi-crystalline thermoplastic polymers. Sundstrom and Lo [51] showed, that it can also be applied to the plastication of amorphous polymers by introducing a proper softening temperature $\vartheta_{m}$. In this simplified approach, the viscosity of the melt film is considered as a Newtonian fluid independent of temperature. Then, the melting rate per unit length $w_{L}$ is

(5) $$w_{L}=\sqrt{\frac{V_{0}\rho_{m}\left[k_{m}\left(\vartheta_{0}-\vartheta_{m}\right)+\frac{\eta }{2}\;V_{0}^{2}\right]W_{drag}}{2\lambda^{**}}\;}.$$

Here, $V_{0}$ is the velocity of the hot moving surface, $\rho_{m}$ is the density of the melt, $k_{m}$ is the heat conductivity of the melt, $\vartheta_{0}$ is the temperature of the moving surface, $\vartheta_{m}$ is the melting temperature of the polymer, η is the mean viscosity of the melt film and $W_{drag}$ is the width of the solid bed. The “2” in the denominator considers the assumption, that 50% of the energy can be used to melt the material, while the rest is transported out of the system by drag flow. $\lambda^{**}$ is the specific enthalpy needed to heat and melt the material

(6) $$\lambda^{**}=\lambda +c_{s}\left(\vartheta_{m}-\vartheta_{s0}\right)+c_{m}\left(\vartheta_{0}-\vartheta_{m}\right)\bar{\theta }.$$

Here λ is the specific heat of fusion, $c_{s}$ is the specific heat capacity of the solid material, $\vartheta_{s0}$ is the initial temperature of the solid material, $c_{m}$ is the specific heat capacity of the melt and $\bar{\theta }$ is the dimensionless temperature which can be simplified to Equation (7) if a Newtonian fluid is considered

(7) $$\bar{\theta }=\frac{2}{3}+\frac{Br}{12}.$$

In this equation, Br is a modified Brinkman number

(8) $$Br=\eta \frac{V_{0}^{2}}{k_{m}\left(\vartheta_{0}-\vartheta_{m}\right)}.$$

The solution of Equations (5)–(8) is an iterative calculation, as the mean melt film thickness and the mean temperature in the melt film are not known at the beginning but needed to calculate the viscosity η. The mean temperature $\bar{\vartheta_{Film}}$ of the melt film is calculated from the temperature of the surface $\vartheta_{0}$, the melting temperature $\vartheta_{m}$ and the dimensionless temperature $\bar{\theta }$

(9) $$\bar{\vartheta_{Film}}=\vartheta_{m}+\bar{\theta }\left(\vartheta_{0}-\vartheta_{m}\right).$$

The mean melt film thickness $\bar{\delta }$ is

(10) $$\bar{\delta }=\frac{\delta_{0}+\delta_{max}}{2},$$

where $\delta_{max}$ is the maximum melt film thickness

(11) $$\delta_{max}=\delta_{0}\sqrt{\left(4+2Br\right)}.$$

The term $\delta_{0}$ is

(12) $$\delta_{0}=\sqrt{\frac{k_{m}\left(\vartheta_{0}-\vartheta_{m}\right)W_{drag}}{\lambda^{*}\rho_{m}V_{0}}},$$

where $\lambda^{*}$ is the energy needed to heat the solid material to the melting point including the heat of fusion λ

(13) $$\lambda^{*}=\lambda +c_{s}\left(\vartheta_{m}-\vartheta_{s0}\right).$$

The viscosity is calculated from the mean temperature of the melt film ${\bar{\vartheta }}_{Film}$ and the mean shear rate $\left|\bar{\dot{\gamma }}\right|$, which is calculated from the mean melt film thickness $\bar{\delta }$ and from the velocity of the surface $V_{0}$

(14) $$\left|\bar{\dot{\gamma }}\right|=\frac{V_{0}}{\bar{\delta }\;}.$$

In this work, the shear rate and temperature dependency of viscosity is described by a Power-law approach

(15) $$\eta \left(\dot{\gamma },\vartheta \right)=a_{\vartheta ,c}K{\left|\dot{\gamma }\right|}^{n-1}.$$

Here K is the consistency at the melting temperature $\vartheta_{m}$, n is the power law index and $a_{\vartheta ,c}$ is the temperature shift factor described by an exponential law

(16) $$a_{\vartheta ,c}\left(\vartheta \right)=e^{-a\left(\vartheta -\vartheta_{m}\right)}.$$

In this equation, ϑ is temperature, $\vartheta_{m}$ is the melting temperature or the introduced softening temperature in the case of amorphous polymers and a is a material parameter. The shear stress $\tau_{calc}$ is calculated from the mean shear rate $\left|\bar{\dot{\gamma }}\right|$ and the viscosity η

(17) $$\tau_{calc}=\eta \left|\bar{\dot{\gamma }}\right|.$$

To apply this model, the following steps must be taken:

1. Calculation of the specific enthalpy needed to heat and melt the material $\lambda^{*}$ from Equation (13).
2. Assuming initial values for the mean temperature of the melt film ${\bar{\vartheta }}_{Film}$ and the mean shear rate $\dot{\gamma }$ in the melt film.
3. Calculation of the viscosity of the melt film from the mean temperature and the mean shear rate according to Equations (15) and (16).
4. Calculation of the thermal conductivity $k_{m}$ of the melt (if temperature related data is available).
5. Calculation of the modified brinkman number Br from Equation (8).
6. With this value, the dimensionless temperature $\bar{\theta }$ (Equation (7)), the mean temperature of the melt film ${\bar{\vartheta }}_{Film}$ (Equation (9)) and the terms $\delta_{0}$, $\delta_{max}$ and $\bar{\delta }$ (Equations (12), (11) and (10)) are calculated, respectively.
7. The mean shear rate $\dot{\gamma }$ in the melt film can be calculated from the velocity of the surface $V_{0}$ and from the mean melt film thickness $\bar{\delta }$ from Equation (14).
8. Now, one must go back to step 3 and apply the mean temperature of the melt film ${\bar{\vartheta }}_{Film}$, calculated in step 6, and the mean shear rate $\left|\bar{\dot{\gamma }}\right|$ in the melt film, calculated in step 7. These steps must be repeated till the results converge.
9. The specific enthalpy needed to heat and melt the material $\lambda^{**}$ is calculated from Equation (6).
10. The melting rate per unit length $w_{L}$ is calculated from Equation (5).

### 2.6. Comparison of Experimental Results and Calculations

In the experiment, the measured melting rate per area ($w_{A,meas}$) with the unit kg m^−2^s^−1^, is evaluated from the velocity of the piston ($v_{p}$) and the bulk density ($\rho_{bulk}$) of the material by Equation (1). In Tadmor´s drag induced melt removal model, the melting rate per unit length $w_{L}$ has the unit kg m^−1^s^−1^ in Equation (5) which means, that it is not normalized to the width of drag flow $W_{drag}$. For means of a better comparability we introduce the “calculated melting rate per area” $w_{A,calc}$ with the unit kg m^−2^s^−1^, which is obtained by dividing the melting rate per unit length $w_{L}$ through the width of drag flow $W_{drag}$

(18) $$w_{A,calc}=\frac{w_{L}}{W_{drag}}.$$

### 2.7. Analysis of the Melting Sequence

In addition, a method was developed to analyze whether the different materials of a polymer mixture melt simultaneously or rather consecutively. Melting experiments with pure red colored HDPE, pure blue colored PP-H, grade B and different mixtures of them were conducted. The color of the melt film which was dragged out of the sample chamber was evaluated to determine the amount of each polymeric material. Most of these optical analyses were done once, the reproducibility was verified with the mixture of 50 wt.% PP-H, grade B (colored blue) and 50 wt.% HDPE (colored red).

## 3. Materials

The experiments were conducted using five different materials (see Table 3). Both polypropylene-homopolymer (PP-H) resins, the high-density-polyethylene (HDPE), and the polymethylmethacrylate (PMMA) are injection-molding grades and offer a good flowability. The PP-H grades are very similar. Type A was replaced by type B, as the former type A is no longer available. The polypropylene-block-copolymer (PP-B), which is used for pipe extrusion, exhibits a high molecular weight and a low melt flow rate. The polyamide 6 (PA6) is a general-purpose extrusion grade with a low viscosity. The properties and characteristics of those polymeric materials, which are listed in Table 3 and Table 4, were obtained from material datasheets and from experiments. The rheological experiments were carried out using a high-pressure capillary rheometer Rheograph 6000 from Goettfert, Buchen, Germany. Two capillaries with a diameter of 1 mm and a length of 10 and 20 mm were used in the experiments. The range of the apparent wall shear rate was 2.8 to 1400 s^−1^. After applying Bagley correction [55] and Weißenberg Rabinowitsch correction [56] the true viscosity curves were obtained, which were approximated by power law. The thermal analysis was done using a differential scanning calorimetry DSC 3 from Mettler Toledo, Columbus, OH, USA, with a sample weight of 10 ± 1 mg and a heating rate of 5 K min^−1^. The second heating cycle was considered for the evaluation of the thermal parameters of the polymeric materials.

Polypropylene and HDPE are non-polar materials, while PMMA and PA6 are polar materials (see Table 5). The mixture of PP-H and PMMA is incompatible. The mixture of PP-H and PA6 is also rather incompatible, but less incompatible than PP-H and PMMA. The mixture of PP-H and HDPE is described as rather compatible [57,58]. The mixture of PP-H and PP-B should be compatible. The viscosity of PP-B is about three times higher than that of PP-H, that of the other material is about two times higher, depending on the temperature and the shear rate (data not shown).

## 4. Results and Discussion

### 4.1. Bulk Density of the Material Mixtures

As Equation (1) shows, the bulk density is necessary to calculate the melting rate. Kneidinger et al. [61] reported, that the bulk density of polymer bulk solids is dependent on the specific density and the temperature dependent mechanical behavior of the material itself, on the shape and the dimensions of the different bulk materials, on the sample chamber geometry and on the applied normal stress.

**Figure 4:**
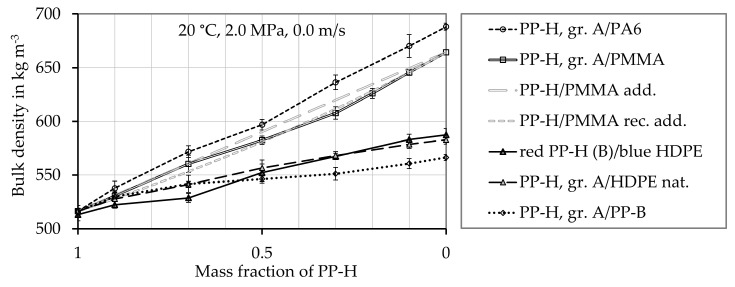
Density of the mixtures of the bulk material. The symbols show experimentally determined values, the black lines connect these experimentally determined values. The gray lines show the density of the PP-H/PMMA mixture calculated by the additivity and the reciprocal additivity relationship model.

The bulk densities of the pure materials PP-H (grade A and B), PP-B, virgin HDPE (natural), blue colored HDPE, PMMA, and PA 6 are 516 kg m^−3^, 566 kg m^−3^, 582 kg m^−3^, 587 kg m^−3^, 664 kg m^−3^, and 688 kg m^−3^, respectively. The experimentally determined bulk density values of the polymer mixtures are given in Figure 4. The density of the mixtures $\rho_{bulk,mix}$ seems to be almost linearly depending on the mass fraction $\xi_{mass,PP-H}$ of PP-H. A linear dependence on the mass fraction $\xi_{A}$ of material A is described by a mass fraction weighted mixing rule of the bulk density of the pure materials A $\rho_{bulk,A}$ and B $\rho_{Bulk,B}$

(19) $$\rho_{bulk,mix,add}=\;\xi_{A}\rho_{Bulk,A}+\left(1-\;\xi_{A}\right)\rho_{Bulk,B}.$$

Equation (19) is only a first approach to calculate the bulk density of the polymer mixtures. Under the prerequisite that the dimensions of the granules are similar and the pressure is rather low, the specific volume of the polymer bulk material mixture $v_{spec,bulk,mix}$ is calculated from the sum of the occupied volume and from the sum of the masses of the materials. In this manner, the specific volume of the mixture of bulk materials $v_{spec,bulk,mix}$ can be calculated from the specific volume of bulk material A and B $v_{spec,bulk,A/B}$ and from the mass fraction of material A and B $\xi_{A/B}$

(20) $$v_{spec,bulk,mix}=\;\xi_{A}v_{spec,bulk,A}+\left(1-\;\xi_{A}\right)v_{spec,bulk,B}.$$

Subsequently, the specific volumes are substituted by the reciprocal values of the corresponding densities. Such an relationship is generally referred to it as “reciprocal additivity relationship” or “inverse additivity relationship” (compare Han [62], Wilczyński et al. [27]). Now, the density of the polymer mixture $\rho_{bulk,mix,rec.add}$ is calculated from the sum of the reciprocal values of the bulk density values of the pure materials multiplied with each mass fraction

(21) $$\frac{1}{\rho_{bulk,mix,rec.add}}=\frac{\xi_{A}}{\rho_{Bulk,A}}+\frac{1-\;\xi_{A}}{\rho_{Bulk,B}}.$$

When more than two materials are involved, the generalized form of Equation (21) can be applied to calculate the bulk density of polymer mixtures $\rho_{Bulk,mix,rec.add}$

(22) $$\frac{1}{\rho_{bulk,mix,rec.add}}=\sum_{i=1}^{n_{m}}\frac{\xi_{i}}{\rho_{Bulk,i}}\;,$$

where $n_{m}$ is the number of materials involved, $\xi_{i}$ is the mass proportion of material i and $\rho_{Bulk,i}$ is the bulk density of material i. Both, the additivity and the reciprocal additivity relationship are exemplarily plotted for the mixture of PP-H and PMMA in Figure 4. In this case, the additivity relationship (Equation (19)) fits the presented data slightly better for lower mass fractions of PP-H, while the reciprocal additivity relation fits it better for higher mass fractions (Equation (22)). In general, the additivity relationship (Equation (19)) fits the presented data slightly better than the reciprocal additivity relation (Equation (22)) (data not shown).The difference between the mixing rules for the bulk density is small, when the bulk density of the pure materials does not differ significantly.

### 4.2. Melting Rates and Shear Stresses of the Pure Materials

**Figure 5:**
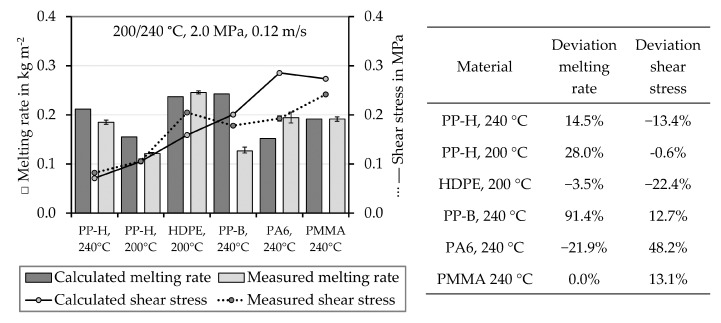
Measured and calculated melting rates of the pure bulk materials and the deviations between the experimentally obtained and the calculated melting rate and shear stress values. PP-H stands for PP-H, grade A. Positive deviations mean that the calculated value is higher than the experimental determined value.

At first, the melting behavior of pure bulk materials is analyzed and compared to Tadmor’s computations. The experimentally obtained values of the melting rates $w_{A,meas}$ and the shear stress $\tau_{meas}$ of the pure bulk materials are compared to the melting rates $w_{A,calc}$ and shear stress values $\tau_{calc}$ calculated from Equations (17) and (18), respectively. These values and the deviations between the calculation and the experimental results are shown in Figure 5. The calculated melting rate of PP-H, grade A is slightly higher than the experimentally obtained value at a temperature of 240 °C. At lower temperature (200 °C) the model predicts a value clearly higher than the experimentally acquired one. The shear stress is slightly underestimated by the calculation at 240 °C. At 200 °C the calculated and the experimentally obtained value are almost equal.

The calculated melting rate for HDPE is confirmed by the experiment (2.7% deviation), but the experimentally determined shear stress was significantly higher than the calculated one. The calculated melting rate of PP-B is nearly twice the experimentally determined value, while the shear stress was slightly overestimated. The melting rate of PA 6 is significantly underestimated by the calculation, while the shear stress is overestimated by nearly 50%.

The application of Tadmor´s melting model to the amorphous PMMA is doubtful. As Sundstrom and Lo [51] showed, it is possible by employ a proper melting temperature $\vartheta_{m}$ which is an assumed softening temperature in this case. Their approach was applied in this work, which means that the applied melting temperature $\vartheta_{m}$ (assumed softening temperature) was optimized until the calculated melting rate equals the experimentally determined value. The shear stress was overestimated by the calculation by 13%. As known from Zitzenbacher and Brunner [63] and Zitzenbacher et al. [64] this PMMA grade tends to slip at the wall, which could explain the overestimation of the shear stress.

As the comparisons between the melting experiment and the calculations show, the drag induced melt removal model indeed predicts the melting rates and shear stresses of some pure polymeric materials well, but not in all cases. For this reason, this model is not applied to determine the melting behavior of the polymer mixtures. As a first solution an approach is proposed to determine the melting behavior of polymer mixtures based on the experimentally determined values of the pure materials.

### 4.3. Melting Rates and Shear Stresses of Material Polymer Mixtures

**Figure 6:**
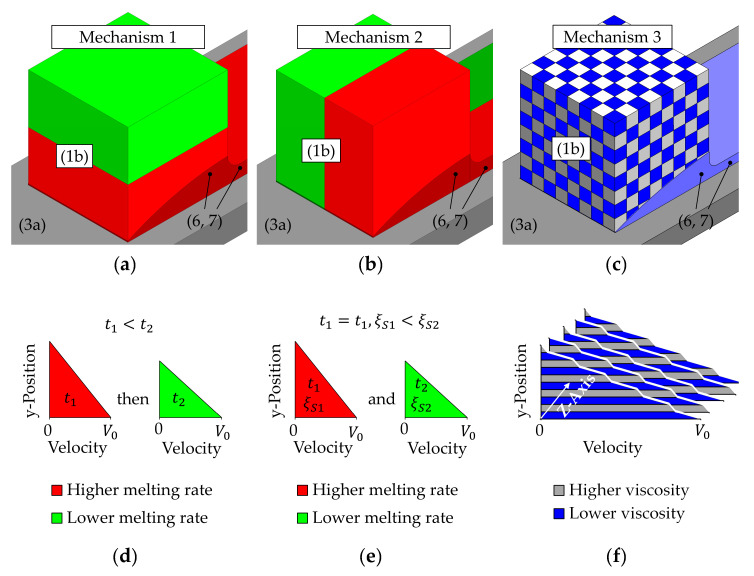
Schematic depiction of the three considered melting mechanisms. The consecutive numbering is consistent to Figure 2: (1b) Solid bulk polymer, (3a) hot moving infinite plate, (6,7) melt film in the sample chamber and outside of it; (**a**–**c**) schematic arrangement of the materials, (**d**–**f**) schematic depiction of the velocity profiles. (**a**,**d**) Consecutively melting of the materials; (**b**,**e)** Simultaneous melting of the materials with homogeneous melt films. (**c**,**f**) Simultaneous melting of the materials with a layered melt film.

**Figure 7:**
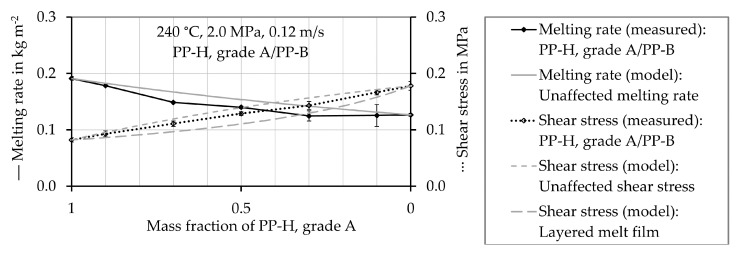
Measured and estimated values of the melting rate and shear stress of the mixture of PP-H, grade A and PP-B at a temperature of 240 °C.

**Figure 8:**
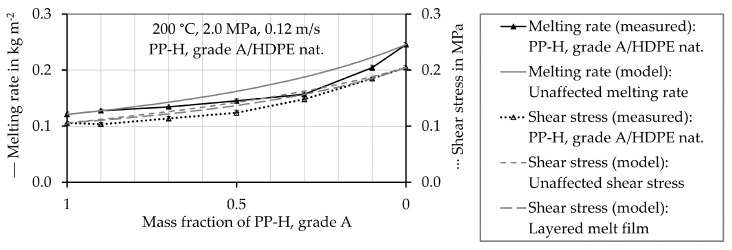
Measured and estimated values of the melting rate and shear stress of the mixture of PP-H, grade A, and HDPE at a temperature of 200 °C.

**Figure 9:**
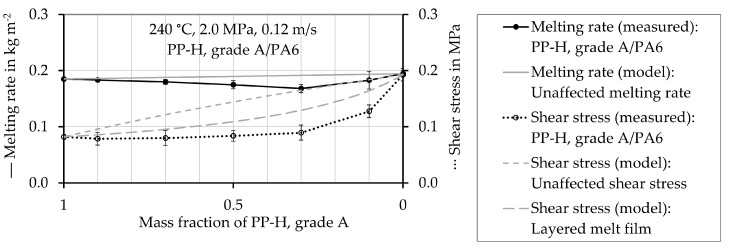
Measured and estimated values of the melting rate and shear stress of the mixture of PP-H, grade A, and PA6 at a temperature of 240 °C.

**Figure 10:**
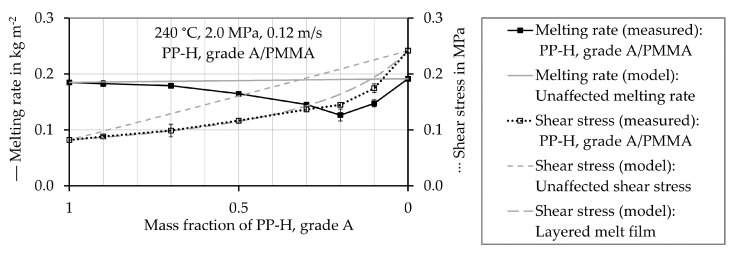
Measured and estimated values of the melting rate and shear stress of the mixture of PPH, grade A, and PMMA at a temperature of 240 °C.

The melting behavior of the polymer mixtures is analyzed using a melting experiment similar to the drag induced melt removal principle. In addition, the melting behavior of the mixtures is predicted based on the experimentally determined values of the pure materials. The results of the melting experiments with the polymer mixtures are compared to relational functions based on the melting behavior of pure bulk solids in Figures 7–10. For polymer mixtures of two or more materials, three melting mechanisms (see Figure 6) are proposed, which are considered with relational functions:

1. The first mechanism assumes a complete consecutive melting of both materials, so that the material with the lower melting rate is melted after the material with the higher melting rate (Figure 6a,d). In this case, the slower melting material requires more time to melt ($t_{2})$ than the other one ($t_{1})$; but the melting of each material is unaffected by the other material(s).
2. The second mechanism considers a complete simultaneous melting of the materials. It is supposed that different homogeneous melt films exist side by side at the same time (Figure 6b,e). If the materials melt simultaneously, the slower melting material requires a bigger proportion ($\xi_{s2}$) of the contact area than the other one ($\xi_{s1}$); but the area-related melting rate of each material is again unaffected by the other material(s).
3. The third mechanism also assumes simultaneous melting of the polymers. In this case, a fine structured, layered melt film consisting of alternating materials over the thickness of the melt film (in y – direction) is assumed (Figure 6c,f). In this case, the melting rate of each material is affected by the other material(s).

If one of the two unaffected mechanisms occurs, both the mean melting rate ${\bar{w}}_{a,mix,unaffected}$ and the mean shear ${\bar{\tau }}_{mix,unaffected}$ stress of the mixture can be determined from the values of the pure materials. However, if the third mechanism occurs, the mean melting rate ${\bar{w}}_{a,mix}$ and the mean shear stress ${\bar{\tau }}_{mix}$ are affected by this mechanism. This influence can be considered by the synergy factors $f_{syn,w}$ and $f_{syn,\tau }$. Synergy factors greater than one represent an increase of a value and synergy factor lower than one signify a reduction. The mean melting rate of a polymer mixture ${\bar{w}}_{a,mix}$ is calculated from the mean unaffected melting rate ${\bar{w}}_{a,mix,unaffected}$ of this mixture and from the synergy factor of the melting rate $f_{syn,w}$

(23) $${\bar{w}}_{a,mix}={\bar{w}}_{a,mix,unaffected}\;f_{syn,w}.$$

The mean unaffected melting rate ${\bar{w}}_{a,mix,unaffected}$ of a mixture of $n_{m}$ materials with the mass proportions $\xi_{i}$ and the melting rate $w_{a,i}$ of the pure material i is calculated from the sum of the materials and the sum of the time needed to melt them, so a reciprocal additivity relation has to be applied once again

(24) $$\;\frac{1}{{\bar{w}}_{a,mix,unaffected}}\;=\sum_{i=1}^{n_{m}}\frac{\xi_{i}}{w_{a,i}}.$$

The synergy factor of the melting rate $f_{syn,w}$ has to be determined experimentally. It is calculated from the measured melting rate $w_{A,meas}$ and from the mean unaffected melting rate ${\bar{w}}_{a,mix,unaffected}$ of this mixture

(25) $$f_{syn,w}=\frac{w_{a,meas}}{{\bar{w}}_{a,mix,unaffected}}.$$

A synergy factor lower than one represents an anti-synergetic behavior which means that the mean melting rate of a polymer mixture ${\bar{w}}_{a,mix}$ is lower than the mathematically predicted mean unaffected melting rate ${\bar{w}}_{a,mix,unaffected}$.

A theoretical prediction of the shear stress of a polymer mixture $\tau_{mix}$ is more complex. The shear stress should typically be between the two values of the pure materials. If a layered melt film (theoretical melting mechanism three, affected mechanism) is considered and slip between the layers of different materials occur like observed (compare [65,66,67,68,69,70]), the shear stress could be lower as well. Moreover, the shear stress will be reduced as the lower viscous material is sheared much more than the rest of the film and is heated by dissipation. This increase of the temperature further reduces the viscosity and the shear stress of the considered layered film. Vice versa, a reduction of the melting rate will cause a reduction of the melt film thickness, which can imply an increase of the shear stress.

As a first solution, it is proposed to calculate the mean shear stress of a mixture of materials ${\bar{\tau }}_{mix}$ from the mean unaffected shear stress ${\bar{\tau }}_{mix,unaffected}$ of a mixture and from the corresponding synergy factor of the shear stress $f_{syn,\tau ,}$

(26) $${\bar{\tau }}_{mix}\;={\bar{\tau }}_{mix,unaffected}\;f_{syn,\tau }\;.$$

This mean unaffected shear stress ${\bar{\tau }}_{mix,unaffected}$ of a mixture is the theoretically mean value of the shear stress of a polymer mixture considering one of the two unaffected mechanisms. It results from the consideration that the melting process of the different materials is independent (“unaffected”) of each other material(s)

(27) $${\bar{\tau }}_{mix,unaffected}=\sum_{i=1}^{n_{m}}\xi_{res,i}\tau_{i},$$

where $\xi_{res,i}$ is the “resource proportion” which considers that, e.g., an amount of 50% of the slower melting material requires more “resources” than 50% of the faster melting material. If the materials are melted consecutively (mechanism one), the “resources” are expressed by the proportion of time needed to melt the material. In the case that the materials melt simultaneously (mechanism two), the “resources” are the required surface proportion. The resource proportion $\xi_{res,i}$ is calculated from the mass proportion $\xi_{i}$ and the melting rate of the pure material $w_{a,i}$. In addition, the mean unaffected melting rate ${\bar{w}}_{a,mix,unaffected}$ of a mixture is needed to calculate this value

(28) $$\xi_{res,i}=\xi_{i}\frac{{\bar{w}}_{a,mix,unaffected}}{w_{a,i}}.$$

The synergy factor $f_{syn,\tau }$ is calculated from the measured shear stress $\tau_{meas}$ and the mean unaffected shear stress ${\bar{\tau }}_{mix,unaffected}$

(29) $$f_{syn,\tau }=\frac{\tau_{meas}}{{\bar{\tau }}_{mix,unaffected}}.$$

The value of this synergy factor must be determined experimentally. A value lower than one represents an anti-synergetic behavior, which means that the mean shear stress ${\bar{\tau }}_{mix}$ is lower than the mean unaffected shear stress ${\bar{\tau }}_{mix,unaffected}$.

When a layered melt film (mechanism three, Figure 6c,f) is considered, the melting process of the different materials is not independent of each other anymore, it is affected. When a layered film is sheared by pure drag flow, all layers are exposed to the same shear stress τ. The mean shear rate $\left|\bar{\dot{\gamma }}\right|$ is calculated from the shear rate $\left|\dot{\gamma_{i}}\right|$ and the volumetric proportion $\xi_{Vi}$ of the different layers of material

(30) $$\left|\bar{\dot{\gamma }}\right|=\sum_{i=1}^{n_{m}}\xi_{Vi}\left|{\dot{\gamma }}_{i}\right|.$$

In the case of Newtonian materials, the shear rate ${\dot{\gamma }}_{i}$ is calculated from the shear stress τ and the viscosity of the different layers $\eta_{i}$

(31) $$\left|{\dot{\gamma }}_{i}\right|=\frac{\tau }{\eta_{i}}.$$

The mean shear rate $\left|\bar{\dot{\gamma }}\right|$ is calculated from the shear stress τ and the mean viscosity of the layered melt film $\bar{\eta }$

(32) $$\left|\bar{\dot{\gamma }}\right|=\frac{\tau }{\bar{\eta }}.$$

After introducing Equations (31) and (32) in Equation (30) the shear stress τ is found on both sides of the equation and is cancelled out. The mean viscosity $\bar{\eta }$ is calculated from the reciprocal additivity relation of the viscosities $\eta_{i}$ of the involved materials and their volumetric proportions $\xi_{Vi}$.

(33) $$\frac{1}{\bar{\eta }}=\sum_{i=1}^{n_{m}}\frac{\xi_{Vi}}{\eta_{i}}.$$

The resulting shear stress is directly related to the viscosity, so the reciprocal additivity relation can be applied to estimate the shear stress considering a layered melt film (theoretical model three) $\tau_{mix,layered}$ of a mixture of Newtonian materials neglecting the temperature dependence of the viscosity

(34) $$\frac{1}{\tau_{mix,layered}}=\sum_{i=1}^{n_{m}}\frac{\xi_{Vi}}{\tau_{i}}.$$

Here $\tau_{i}$ is the shear stress of the pure material i and $\xi_{Vi}$ is the volumetric proportion of material i, calculated from the melt density of the pure material $\rho_{m,i}$ and from the melt density of the mixture $\rho_{m,mix}$

(35) $$\xi_{Vi}=\xi_{i}\frac{\rho_{m,mix,rec.add}}{\rho_{m,i}}.$$

The melt density of the mixture is denoted as $\rho_{m,mix,rec.add}$ as it is calculated analogously to the density of mixtures of immiscible liquids from the reciprocal additivity relation

(36) $$\frac{1}{\rho_{m,mix,rec.add}}=\sum_{i=1}^{n_{m}}\frac{\xi_{i}}{\rho_{m,i}}.$$

A synergy factor considering a layered melt film $f_{syn,\tau ,layered}$ can be calculated from the measured shear stress $\tau_{meas}$ and from the shear stress considering a layered melt film $\tau_{mix,layered}$ again

(37) $$f_{syn,\tau ,layered}=\frac{\tau_{meas}}{\tau_{mix,layered}}.$$

Those results are not shown, as they do not provide further insight. In Figure 7, Figure 8, Figure 9 and Figure 10 the experimentally determined melting rates of the polymer mixtures are compared to the values estimated by the model describing the mean unaffected melting rate ${\bar{w}}_{a,mix,unaffected}$. Furthermore, experimentally determined shear stress values are compared to the estimated ones. They are received from both, the mean unaffected shear stress ${\bar{\tau }}_{mix,unaffected}$ of a material mixture and the shear stress considering a layered melt film $\tau_{mix,layered}$. The symbols represent the experimentally determined values and the black lines connect these values. The gray lines show the results obtained by calculations using the models. The values are plotted as a function of the proportion of PP-H, starting with pure PP-H (100 wt.%, mass fraction 1) at the left-hand side of the horizontal axis.

Figure 7 shows that the melting rate of pure PP-B (mass fraction PP-H = 0) is lower compared to pure PP-H (mass fraction PP-H = 1), which means that pure PP-B melts slower than pure PP-H. In addition, the shear stress is much higher when testing pure PP-B. All measured melting rates of the polymer mixtures are lower than the theoretically reachable mean unaffected melting rate ${\bar{w}}_{a,mix,unaffected}$ of a mixture calculated from Equation (24). The experimentally determined shear stress values of the mixtures are between the predicted values.

Pure HDPE (mass fraction PP-H = 0) melts faster than pure PP-H (mass fraction PP-H = 1) (see Figure 8). The measured melting rates of the polymer mixtures with 10 or 30 wt.% HDPE are slightly below the theoretically reachable mean unaffected melting rate ${\bar{w}}_{a,mix,unaffected}$. That of a polymer mixtures which contain 50 wt.%, 70 wt.%, and 90 wt.% HDPE respectively exhibit a significantly lower melting rate compared to the theoretically reachable mean unaffected melting rate ${\bar{w}}_{a,mix,unaffected}$. Both approaches for the estimation of the shear stress predict similar values for the polymer mixture PP-H/HDPE because pure HDPE melts faster than pure PP-H and the shear stress of pure HDPE is higher as well. Contrary to these calculations, the measured shear stress values are lower than the calculated ones. In this case the approach considering a layered melt film $\tau_{mix,layered}$, according to Equation (34) represents the smallest deviations from the experimentally obtained shear stress values.

The melting rates of pure PA 6 (mass fraction PP-H = 0) and of pure PMMA (mass fraction PP-H = 0) are close to that of pure PP-H, but the shear stress of PP-H is much lower in both cases (see Figure 9 and Figure 10). The calculations predict a linear dependence of the melting rate on the mass fraction of PP-H for the mixtures PP-H/PA 6 and PP-H/PMMA. In the experiment, first a decrease in the melting rate with decreasing mass fraction PP-H was obtained. After reaching a minimum value, an increase in the melting rate occurred. Especially the mixture PP-H/PMMA exhibits a melting behavior which is completely different to the linear prediction of the mixing rule.

The mean unaffected shear stress ${\bar{\tau }}_{mix,unaffected}$ of a mixture (calculated from Equation (27)) is almost linear as well, but does not fit well in both cases. The approach considering a layered melt film $\tau_{mix,layered}$ (calculated from Equation (34)) results in smaller deviations from the experimentally determined values. It describes the shear stress of the mixture of PP-H/PMMA very well, but still overestimates it in the case of PP-H/PA 6. Zhao, Macosko, Ahonguio et al. [65,66,67,68] reported that interfacial slip between PP-H and PA 6 can reduce the shear stress in the case of a multilayer flow.

**Figure 11:**
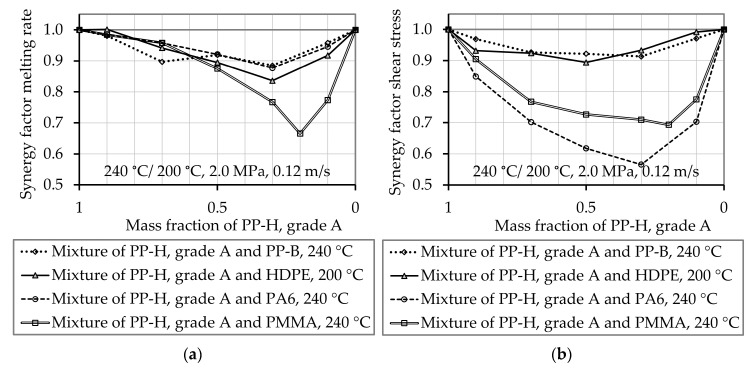
Experimentally determined synergy factors of the melting rate for the mixtures PPH/HDPE, PP-H/PA6, and PP-H/PMMA.

All experimentally determined melting rates of the polymer mixtures are lower than the theoretically predicted values. The synergy factor for the melting rate $f_{syn,w}$, which is calculated according to Equation (24), is 1 for pure materials and smaller than 1 for mixtures (see Figure 11). In the presented experiments, the synergy factor was higher than 0.84, 0.88, 0.88, and 0.67 for the mixtures PP-H/HDPE, PP-H/PA6, PP-H/PP-B, and PP-H/PMMA, respectively. Furthermore, all measured shear stress values are below the mean unaffected shear stress ${\bar{\tau }}_{mix,unaffected}$ of a mixture of materials. The synergy factor for the shear stress, which is calculated according to Equation (29), is 1 for pure materials and below 1 for the mixtures. The mixture PP-H/PA 6 exhibits the lowest synergy factor of all mixtures, reaching a minimum value of 0.566 for a concentration of 30 wt.% PP-H and 70 wt.% PA 6. The lowest observed value for PP-H/PMMA is 0.69 and for PP-H/HDPE as well as PP-H/PP-B the synergy factor is in the range of 0.9. Looking at these results with regard to the compatibility of the compounds, it can be seen that the less compatible compounds (PP-H/PA6 and PP-H/PMMA) deviate noticeably more from the calculated values.

To sum up, it can be stated that when melting polymeric mixtures, both the melting rate and the shear stress are for the major part significantly below the values, which are predicted with mixing rules. An exception are mixtures with small amounts of higher viscous materials. In this case, the melting rate can be estimated with simple mixing rules, but not the shear stress. In general, both the mean melting rate and the mean shear stress of mixtures can be predicted by the proposed equations (Equations (23) and (26)).

### 4.4. Analysis of Extrusion Experiments and Screw-Pullout Experiments

**Figure 12:**
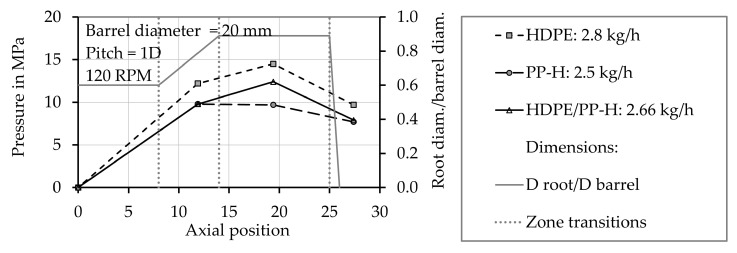
Comparison of the measured pressure profiles when processing pure PP-H, grade A, pure HDPE, and a mixture of 50 wt.% PP-H, grade A and 50 wt.% HDPE.

In order to analyze if the low melting rates of polymer mixtures, which were obtained using the melting experiments, affect the melting behavior in single screw extruders as well, screw pull out experiments were conducted. They were performed using pure HDPE, pure PP-H (grade A) with 2 wt.% blue master batch and a mixture of 50 wt.% HDPE and 50 wt.% PP-H, grade A. The throughput and the pressure profile when processing the pure materials and the mixture are similar (Figure 12). The throughput of HDPE (2.8 kg/h) is higher than that of PP-H (2.5 kg/h). That of the mixture (2.66 kg/h) corresponds nearly exactly to the mean value of the pure materials. The viscosity of HDPE is higher than that of PP-H, so the pressure at the tip is higher as well. After a pressure rise in the feeding and compression zone of the screw the pressure decreases in the metering zone.

**Figure 13:**
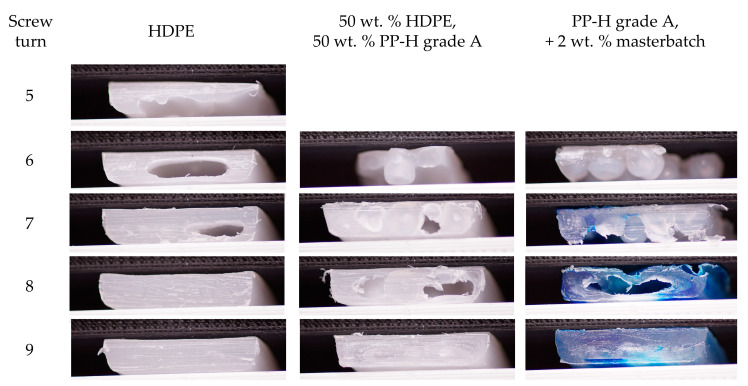
Results of the screw pull out experiments at a screw speed of 120 min^−1^ when processing pure PP-H, grade A, pure HDPE and a mixture of 50 wt.% PP-H, grade A, and 50 wt.% HDPE using a blue masterbatch.

The results of the screw cooling and pull out experiments represented in pictures of the cross section of the filled screw channel are given in Figure 13. The analyses of the melting behavior reveal that HDPE melts earlier and faster than PP-H. The behavior of the mixture is comparable to that of pure PP-H. HDPE is completely melted after 6 turns, PP-H and the mixture after 8 turns. This finding confirms the results of the melting experiments (Figure 8), which shows that a mixture of 50 wt.% PP-H and 50 wt.% HDPE melts only slightly faster than pure PP-H, but significantly slower than pure HDPE.

### 4.5. Optical Analysis of the Melting Sequence

**Figure 14:**
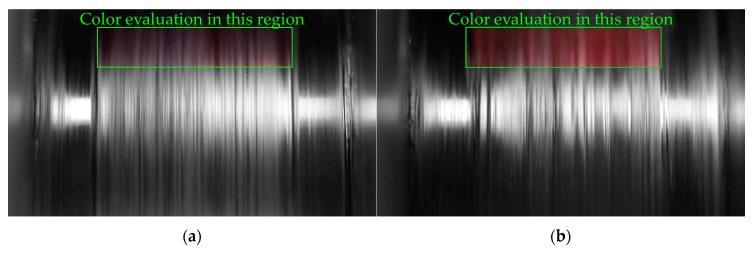
Typical pictures (frames of the video file) captured during melting experiments of two pure polymers. The rectangular shows the analyzed region. The color outside of the analyzed region is not analyzed and greyed out in these depictions. (**a**) Pure blue PP-H, grade B; (**b**) pure red HDPE.

**Figure 15:**
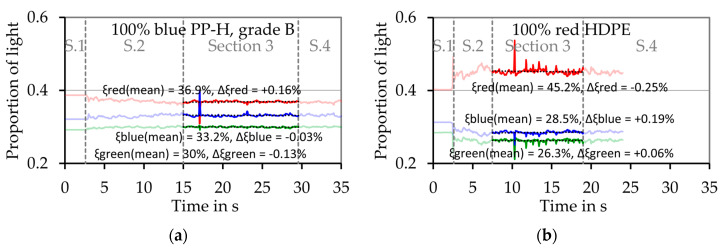
Results of the optical evaluation of the melting experiment for two pure polymers. The dashed line shows the transitions between the four sections. (**a**) Pure blue PP-H; (**b**) pure red HDPE.

Until now, it was assumed that the melting behavior is constant and the different polymers of the mixture melt simultaneously during the whole experiment. To study if this is an acceptable assumption and if the different materials of the mixtures melt simultaneously or rather consecutively, a new method was developed. Therefore, the color of the melt film is measured and analyzed. A video of the melt film is recorded during the melting experiment and evaluated afterwards. The composition of the light $\xi_{RGB}$, which means the proportions of red light $\xi_{R}$, green light $\xi_{G}$ and blue light $\xi_{B}$ for each picture of the video are determined in the “Region of Interest” (ROI), which is shown as green rectangle in the figures. Figure 14 shows two typical pictures captured during the melting pure PP-H, grade B (Figure 14a) and pure HDPE (Figure 14b). The color of the shaft without a melt film is more red than blue (data not shown). The melt film is very thin, so all the analyzed pictures are more red than blue, even for the experiments with pure blue material. Nevertheless, the proportions of red, green and blue light differ significantly depending on the melted material (compare Figure 15b and Figure 18b).

The experiments and the recorded videos are subdivided in four sections to do this analysis. At the beginning of each experiment and each video (Section 1), there is no melt film at the shaft surface. Section 2 is not considered for the evaluation, because the beginning of the melting process is unstable. In this section, the first polymer layer is preheated during the startup of the experiment and the melting rate increases. Section 3 represents the melting process itself. In this part of the experiment, the composition of the light $\xi_{RGB}$ is evaluated and the mean composition of the light ${\bar{\xi }}_{RGB,S3}$ is calculated. Furthermore, a linearization is established and the change of the composition of the light $\Delta \xi_{RGB,S3}$ during this section is calculated. In Section 4, which is not considered in the evaluation, the piston cools the last layer of material because of the direct contact. Furthermore, the penetration of the melt into the solid bed and the drag flow itself is affected here as well.

**Figure 16:**
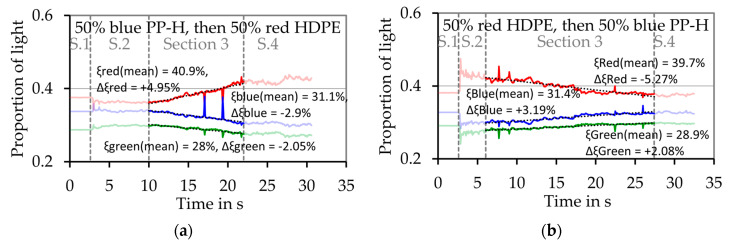
Results of the optical evaluation of the melting experiment for two different polymers. The dashed line shows the transitions between the four sections. (**a**) Filling sequence: First pure blue PP-H, grade B; then pure red HDPE, (**b**) filling sequence: First pure red HDPE, then pure blue PP-H.

Figure 15a,b shows results of melting experiments with pure blue PP-H, grade B, and pure red HDPE respectively. In both cases red light represents the largest proportion of the light, but in case of the pure HDPE this proportion is much bigger than in case of the blue PP-H. For example, in case of pure PP-H (Figure 15a) the mean proportion of red light in Section 3${\bar{\xi }}_{R,S3}$ is 36.9% while the change of the proportion of the red light during this section $\Delta \xi_{R,S3}$ is +0.16%. In case of the pure red HDPE (Figure 15b), ${\bar{\xi }}_{R,S3}$ is 45.2%, so 8.3% higher. This shows that the color of the thin melt film can be detected by the measurement system. In case of the pure materials, the values do not change significant during the evaluation period (Section 3). If the material changes during the experiment, a significant change of the color of the melt film can be detected, as Figure 16 shows. In this case exemplary experiments simulating the first mechanism which assumes a consecutive melting of both materials (Figure 6a) were conducted. Figure 16a shows the results of an experiment were 2.5 g of the blue PP-H were first filled into the sample chamber followed by the same amount of red HDPE. Figure 16b shows the results of an experiment with an inverted filling sequence.

**Figure 17:**
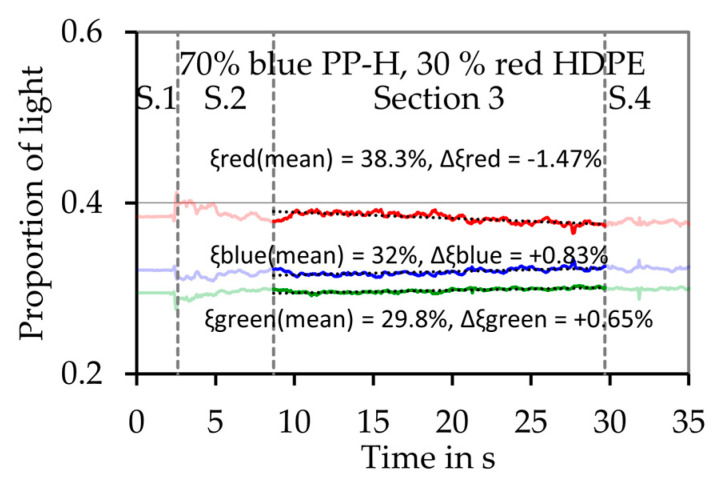
Exemplary result of the optical evaluation of the melting experiment for a mixture of 70 wt.% blue PP-H, grade B and 30 wt.% red HDPE. The dashed line shows the transitions between the four sections.

The experiments conducted with the polymer mixture PP-H/HDPE are analyzed using the same procedure. Figure 17 exemplarily shows the results of an experiment with a mixture of 70% blue PP-H and 30% red HDPE. The mean values of the proportion of light are between the values determined for the pure materials. During the evaluation period (Section 3) the values change significantly. For example, the mean proportion of red light ${\bar{\xi }}_{R,S3}$ is 38.3% and the change of the proportion of the red light during this section $\Delta \xi_{R,S3}$ is −1.47%, which means that at the beginning of Section 3, the proportion of red light is 1.47% higher than at the end. This in turn means, that the proportion of the red HDPE is higher at the beginning of the experiment than at the end.

**Figure 18:**
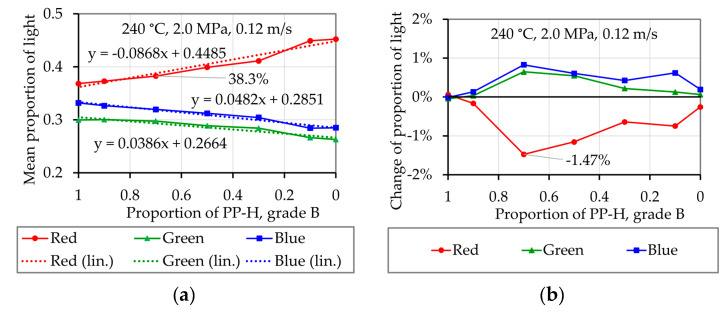
Composition of the light in the melt film for the mixture PP-H/HDPE as a function of the proportion of PP-H, grade B: (**a**) Mean values, the dashed line shows the linearized functions; (**b**) the change of the composition of the light $\Delta {\bar{\xi }}_{RGB,S3}$;

The results of the experiments with the pure materials and the mixtures are combined in Figure 18a,b. The mean composition of the light of section three ${\bar{\xi }}_{RGB,S3}$ is calculated and plotted as a function of the proportion of PP-H, grade B $\xi_{PP-H}$ in Figure 18a. This shows that an increase of 8.68% of the red light represents an increase of the proportion of HDPE of 100%, as well as decrease of blue light of 4.82% and a decrease of green light of 3.86%. These values are denoted as the change of the proportion of light caused by the material $\Delta \xi_{RGB,Mat}$. The change of the proportion of light during the third section $\Delta \xi_{RGB,S3}$ is depicted in Figure 18b. The values of the red-light proportion (${\bar{\xi }}_{R,S3}=\;38.3\%\;\mathrm{and}\;\Delta \xi_{R,S3}=\;-1.47\%)$ of the mixture of 70 wt.% PP-H and 30 wt.% HDPE which where exemplarily shown in Figure 17 are marked in both charts.

The relation of the change of the proportion of light during the third section $\Delta \xi_{RGB,S3}$ and the change of the proportion of light caused by the material $\Delta \xi_{RGB,Mat}$ defines the change of the melted proportion of material during the third section $\Delta \xi_{Mat}$

(38) $$\Delta \xi_{Mat}=\frac{\Delta \xi_{RGB,S3}}{\Delta \xi_{RGB,Mat}}.$$

**Figure 19:**
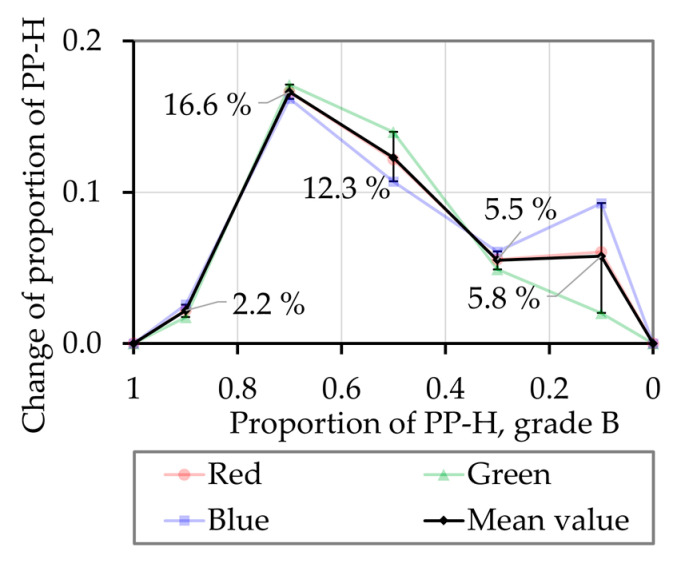
Corrected values of the change of the melted proportion of material $\Delta \xi_{Mat}$ for the mixture PP-H/HDPE as a function of the proportion of PP-H, grade B.

As Figure 15a,b as well as Figure 18b show, a slight change of the proportion of light during the third section $\Delta \xi_{RGB,S3}$ is detected in the experiments with the pure materials as well. The melted portion of material cannot vary when only one material is used. Therefore, these values are considered for calibration of the experiments and corrected to zero. The corrected values of the change of the melted portion of material during the third section $\Delta \xi_{Mat,S3}$ are presented in Figure 19. A value of 0 means that the polymer mixtures are melted completely simultaneously, a higher value tending towards 1 and a lower value tending towards −1 would mean that melting takes place consecutively. When analyzing the mixture 70 wt.% PP-H with 30 wt.% HDPE a value of 16.8% was obtained. This is interpreted that at the beginning of the evaluation the composition of the melt film is 38.4 wt.% HDPE and just 61.6 wt.% PP-H. At the end of the evaluation period, the melt film composition is 21.6 wt.% HDPE and 78.4 wt.% PP-H.

These results reveal that the proportion of melted materials indeed changes during the experiments. A comparison of the experiments with heterogeneous and homogeneous bulk polymers shown in Figure 7, Figure 8, Figure 9 and Figure 10 shows that neither the shear stress nor the melting rate shows a significant increase or decrease during the evaluation period (data not shown).

## 5. Conclusions

In this work the melting behavior of homogeneous polymer and polymer mixtures was analyzed both theoretically and experimentally by model experiments. The results were verified by screw-cooling and -pullout experiments. The model experiments showed that the melting rate and the shear stress can be reduced significantly when polymer mixtures are melted, even if both pure components offer comparable melting rates. Furthermore, it is demonstrated that Tadmor´s drag induced melt removal model predicts the melting rates and shear stresses of some pure polymeric materials well, but not in all cases. The melting behavior and the shear stress of pure materials which melt at rather low shear stress (low viscosity) can be predicted well by the calculations presented by Gogos and Tadmor [6], as observed for the investigated PP-H and HDPE grades. Larger deviations were found when testing the higher viscous PA6 and PP-B. These grades exhibited higher shear stresses. The softening behavior of the amorphous PMMA can be calculated as proposed by Sundstrom and Lo [51], but experiments are necessary to define a proper melting/softening temperature $\vartheta_{m}$. For this reason, a new approach is proposed to determine the melting behavior of polymer mixtures based on the experimentally determined values of the pure materials. The proposed approaches describe the melting rate (Equation (23)) and the shear stress (Equation (25)) of a mixture from mathematically achievable values and from experimentally determined synergy factors. These synergy factors describe the difference between the calculated values and the experimentally determined ones. The mathematically achievable values are obtained by analyses of three theoretical mechanisms. The so-called unaffected melting rate ${\bar{w}}_{a,mix,unaffected}$ of a polymer mixture (Equation (24)) and the unaffected shear stress ${\bar{\tau }}_{mix,unaffected}$ of a polymer mixture (Equation (27)) are calculated from the melting rate and the shear stress of the pure materials. In this work, experimentally determined values of the melting rate and the shear stress of pure materials are applied, but the approaches can also be applied on calculated values. Experiments show that both, the melting rate and the shear stress of mixtures are lower than these calculated “unaffected” values. The determined melting rates are up to 35% lower than the calculated unaffected melting rate ${\bar{w}}_{a,mix,unaffected}$. The determined shear stresses of the mixtures of PP-H/PA6 and PP-H/PMMA are up to 45% lower than the calculated unaffected shear stress ${\bar{\tau }}_{mix,unaffected}$. This huge reduction can be described by slip between the layers. An exception are polymer mixtures with small amounts of higher viscous materials. In this case, the values of the presented mean unaffected melting rate ${\bar{w}}_{a,mix,unaffected}$ and the mean unaffected shear stress ${\bar{\tau }}_{mix,unaffected}$ of a mixture can describe the melting behavior with a high degree of approximation. The reductions are considered by the synergy factors which are lower than one, representing anti-synergetic effects. For example, a reduction of 35% is considered by a synergy factor of 0.65. The synergy factors must be determined experimentally (see Figure 11). Future work will deal with the non-experimental estimation of these synergy factors.

These decreased melting rates can reduce the melting capability of a single screw extruders significantly which was verified by screw-pullout experiments with HDPE, PP-H and a mixture of 50 wt.% HDPE and 50 wt.% PP-H. The axial melting length of pure HDPE in the extruder with a diameter of 20 mm is 6 D, that of PP-H and that of the mixture are 8 D.

Furthermore, a new optical analysis method was developed to evaluate if the different materials are melted simultaneous or rather consecutively in the experiment. Melting experiments with mixtures of blue HDPE and red PP-H were conducted for different mixing ratios. It is shown that these mixtures neither melted completely simultaneously nor completely consecutively. The proportion of HDPE decreases by maximum 16.8% during the observation period, but a significant change of the melting rate or the shear stress during the experiments could not be detected. From this point of view, a simplified simultaneously melting process may be considered for further evaluations.

## Figures and Tables

**Table 1:** Dimensions of the three-section screw.

Dimension	mm	inch	Dimension	Value
Screw diameter D	20.0	0.787	Screw pitch	1 D
Depth feeding zone $h_{1}$	4.0	0.157	Length zone 1 ^1^	8 D
Depth metering zone $h_{3}$	1.1	0.043	Length zone 2 ^2^	6 D
Axial flight width $e^{*}$	2.5	0.098	Length zone 3 ^3^	11 D
Radius pushing flight	1.0	0.039	Tip length	2 D
Radius trailing flight	2.5	0.098	Compression Ratio	3.64

^1^ Feeding zone, ^2^ compression zone, ^3^ metering zone

**Table 2:** Temperature profile.

Unit	Feed throat	Zone 1	Zone 2	Zone 3	Die
°C	85	190	195	200	200
°F	185	374	383	392	392

**Table 3:** Properties of the materials used.

Property	PP-H, Grade A	PP-H, Grade B	PP-B	HDPE	PA6	PMMA
Grade	HD 120MO	HE 125MO	BA 202E	MG 9641B	Ultramid B27E	Plexiglas 7M
Manufacturer	Borealis Polyolefine GmbH, Linz, Austria	Borealis Polyolefine GmbH, Linz, Austria	Borealis Polyolefine GmbH, Linz, Austria	Borealis Polyolefine GmbH, Linz, Austria	BASF SE Ludwigs-hafen, Germany	Röhm GmbH, Darmstadt Germany
Processing ^1^	injection molding	injection molding	extrusion	injection molding	extrusion	injection molding
Properties ^1^	good flow properties	good flow properties,	High viscosity	good flow properties	low viscosity	good flowability
Color	natural/ orange	blue	opaque	natural/red	opaque	transparent
Shape	spheroidal/ cylindrical	cylindrical	lenticular	spherical/ cylindrical	spheroidal	cylindrical

^1^ Information from the datasheets. Abbreviations: PP-H—polypropylene homopolymer; PP-B—polypropylene block copolymer; HDPE—high-density polyethylene; PA6—polyamide 6; PMMA—polymethylmethacrylate.

**Table 4:** Characteristics of the materials used.

Property	Unit	PP-H, Grade A	PP-H, Grade B	PP-B	HDPE	PA6	PMMA
Solid density ^1^ $\rho_{s}$	$\frac{\mathrm{kg}}{m^{3}}$	908	905	900	964	1183	1190
Melt density ^2^ $\rho_{m}$	$\frac{\mathrm{kg}}{m^{3}}$	719	716	710	776	1016	1060
Heat transfer coefficient of the melt ^2^ $\lambda_{m}$	$\frac{W}{\mathrm{mK}}$	0.160	0.161	0.216	0.218	0.217	0.181
Heat capacity of the solid ^3^ $c_{p,s}$	$\frac{kJ}{\mathrm{kgK}}$	1.89	1.89	1.86	1.95	1.90	1.40
Heat capacity of the melt ^3^ $c_{p,m}$	$\frac{kJ}{\mathrm{kgK}}$	2.38	2.36	2.25	2.25	1.95	1.89
Heat of fusion ^3^ λ	$\frac{kJ}{\mathrm{kg}}$	105	105	68.5	199	98	-
Consistency K	$\frac{{\mathrm{Ns}}^{n}}{m^{2}}$	13,502	9246	59,402	9940	7838	60,861
Power law index n		0.324	0.376	0.263	0.486	0.504	0.239
Variation by temperature a	$\frac{1}{K}$	0.00994	0.00817	0.00931	0.00950	0.0137	0.0194
Melting temperature $\vartheta_{m}$	°C	167.93	167.58	165	132	224	223 ^4^

^1^ Information from the datasheets,^2^ Measured with high-pressure capillary rheometer,^3^ Measured with differential scanning calorimetry, ^4^ Applied softening temperature, estimated value.

**Table 5:** Surface tension of the general types of materials used (literature data, Unit Nm^−1^).

Property	PP-H	HDPE	PA6	PMMA
Source	[59]	[59]	[60]	[59]
Surface tension	27.1–42.4	34.6–35.7	38–60	40.3–51.3
Disperse part	25.8–42.1	34.5–35.0	17–30	25.7–39.6
Polar part	0.3–1.3	0.1–0.7	21–30	11.6–14.6
